# Microwave Irradiation Effect on the Dispersion and Thermal Stability of RGO Nanosheets within a Polystyrene Matrix

**DOI:** 10.3390/ma7075212

**Published:** 2014-07-18

**Authors:** Edreese H. Alsharaeh, Ali A. Othman, Mohammad A. Aldosari

**Affiliations:** 1 Department of Chemistry, Alfaisal University, P.O. Box 50927, Riyadh 11533, Saudi Arabia; E-Mail: aothman@alfaisal.edu; 2 King Abdulaziz City for Science & Technology (KACST), National Nanotechnology Research Center (NNRC), P.O. Box 6086, Riyadh 11442, Saudi Arabia; E-Mail: aldosari@kacst.edu.sa

**Keywords:** graphene, graphene oxide, polymer, styrene, MWI

## Abstract

Polystyrene-reduced graphene oxide (PSTY/RGO) composites were prepared via the *in situ* bulk polymerization method using two different preparation techniques. The general approach is to use microwave irradiation (MWI) to enhance the exfoliation and the dispersion of RGO nanosheets within the PSTY matrix. In the first approach, a mixture of GO and styrene monomers (STY) were polymerized using a bulk polymerization method facilitated by microwave irradiation (MWI) to obtain R-(GO-PSTY) composites. In the second approach, a mixture of RGO and STY monomers were polymerized using a bulk polymerization method to obtain RGO-(PSTY) composites. The two composites were characterized by FTIR, ^1^ H-NMR, XRD, SEM, HRTEM, TGA and DSC. The results indicate that the composite obtained using the first approach, which involved MWI, had a better morphology and dispersion with enhanced thermal stability, compared with the composites prepared without MWI. Moreover, DSC results showed that the *T*_g_ value of the composites after loading the RGO significantly increased by 24.6 °C compared to the neat polystyrene.

## 1. Introduction

Graphene is known as the thinnest two-dimensional graphitic carbon (sp^2^-bonded carbon sheet) material and is one atom in thickness [1,2,3]. Graphene has recently attracted much interest as a filler for the development of new composites [4,5,6,7,8,9,10,11,12,13,14]. Its extraordinary structural, mechanical, thermal, optical and electrical properties make graphene an excellent two-dimensional filler material for polymer composites for applications in many technological fields [4,5,6,7,8]. Good dispersion is crucial for achieving the desired enhancement in the final physical and chemical properties of the composites [2]. However, one of the challenges is achieving the good dispersion of the nanoscale filler in the composites, especially for graphene, which has a strong tendency to agglomerate through the intrinsic van der Waals forces and π–π stacking, due to its hydrophobic nature [15]. Various techniques have been developed for the synthesis of these composite structures, including solution mixing, melt blending and *in situ* polymerization [16,17,18,19,20,21].

Recently, a novel approach for the production of reduced graphene oxide nanosheets (RGO) via the reduction of GO, which facilitated by microwave irradiation (MWI), is reported, the resulting product is composed of graphene sheets with polar functional groups, even after the reduction [22]. GO has an affinity for polar solvents and polymers [23]. This affinity makes GO an important intermediate in the preparation of RGO/polymer composites via chemical reduction [21]. In MWI, dielectric heating energy is transferred directly to the reactants, and the energy is supplied to the molecules faster than they are able to relax, creating high instantaneous temperatures that increase the yield and quality of the product [13].

In earlier work by the authors, [24] polymethyl methacrylate (PMMA/RGO) composites were successfully prepared by the *in situ* bulk polymerization via MWI. Experimental results by X-ray diffraction (XRD), Raman spectroscopy and thermogravimetric analysis (TGA) indicated the successful incorporation of the RGO within the polymer matrix. High resolution transmission electron microscopy (HRTEM) results supported the assumption that the incorporation of the RGO in PMMA/RGO composites was due to exfoliation of the filler layers. In addition, the results indicate that the composite obtained using our experimental approach, which involved MWI, had a better morphology and dispersion with enhanced thermal stability, compared with the composites prepared without MWI.

In this work, we report the preparation of polystyrene/RGO composites (PSTY/RGO). Polystyrene (PSTY) is an aromatic polymer and among the most important polymers that is commonly used in a variety of products, ranging from food packaging, thin film electronics and a variety of other applications [22,23,25,26]. Several studies have reported the successful incorporation of GR nanosheets into the PSTY matrix with different preparation techniques using various sources of GR preparation [6,7,8]; however, there is no published reports on preparing such composites using the MWI method to functionalized RGO sheets within a polystyrene matrix by the *in situ* bulk polymerization method. Herein, we report two different synthesis techniques of (PSTY/RGO) composites. In the first approach, a mixture of GO and styrene monomers (STY) were polymerized using a bulk polymerization method; after the addition of a reducing agent, hydrazine hydrate (HH), the product was reduced using a conventional microwave to obtain R-(GO-PSTY) composites. In the second approach, a mixture of RGO, which was produced via MWI, and STY monomers were polymerized using a bulk polymerization method to obtain RGO-PSTY composites.

In addition, it is interesting to investigate the effect of MWI on the type of interaction and the thermal stability of the same filler (RGO nanosheets) with a different type of polymer matrix, methyl methacrylate (hydrophilic), compared to styrene (hydrophobic). The results suggest that MWI may affect the type of dispersion (intercalation *vs.* exfoliation) and interfacial interactions between the RGO nano-filler sheets within the polymer matrix. The experimental results suggest that the de-lamination and exfoliation of the RGO sheets was enhanced and improved with the MWI approach and showed better dispersion of the RGO filler within the polymer matrix, hence enhancing the thermal properties of the RGO/PSTY composites.

## 2. Results and Discussion

**Figure 1:**
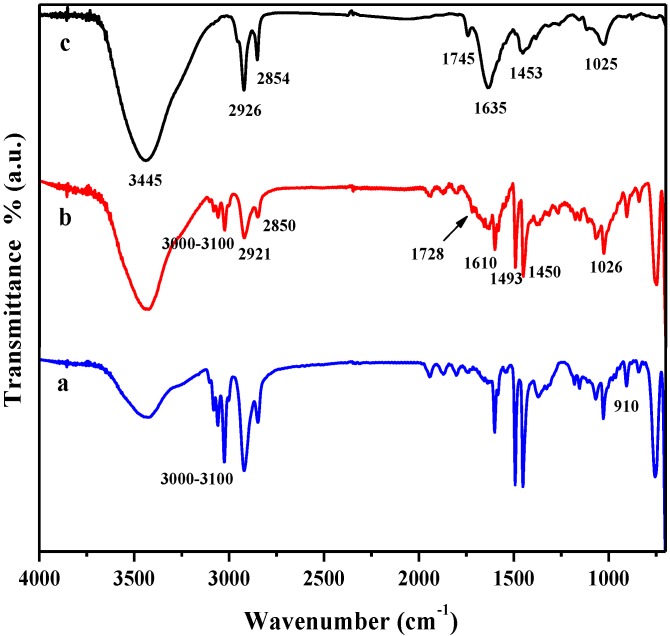
FTIR spectra of (a) neat polystyrene (PSTY); (b) reduced graphene oxide (RGO)-PSTY and (c) R-(GO-PSTY) composites.

FTIR spectral analysis was performed to confirm the chemical structure of all of the RGO/PSTY composites. Figure 1 summarizes the FTIR spectra of the neat PSTY, RGO-PSTY and R-(GO-PSTY) composites. The characteristic FTIR features of GO and RGO have been reported previously by the authors [20]. For the neat PSTY (Figure 1a), the spectrum shows the typical characteristic bands for PSTY at 2925, 2850, 1680 to 2000 and 1610 cm^−1^, which correspond to the aliphatic C–H and –CH_2_ and the aromatic C=C stretching, respectively. Finally, four peaks in the region from 3000 to 3100 cm^−1^ correspond to aromatic C–H and =C–H stretching.

For the RGO-PSTY composites (Figure 1b,c), the spectra show the characteristic bands at 3420, 1728 and 1610 cm^−1^ that correspond to the O–H, C=O and C=C groups, respectively. This confirms the presence of RGO within the PSTY matrix. Interestingly, there is an increase of the C=C band intensity, shifted to 1635 cm^−1^ (Figure 1c) in comparison with (Figure 1b), when the microwave was not used; this may suggest that this enhancement in the aromatic region is due to π–π stacking. Moreover, Figure 1c shows that in the aromatic overtones, aromatic C–H and =C–H stretching regions, the bands were reduced in intensity compared to Figure 1b. This might suggest that the π-bonds of PSTY and RGO sheets opened by the MWI; this will induce more electron chain transfer sites and, therefore, will promote more interfacial interactions between RGO nanosheets within the PSTY matrix. Previous studies reported the interaction between GO and polymers, and it has been observed that the absorption bands of polymer/GO (or graphene) composite have a shift of about several wavenumbers compared with the pure polymer, due to the interaction between GO and the polymer [26].

**Figure 2:**
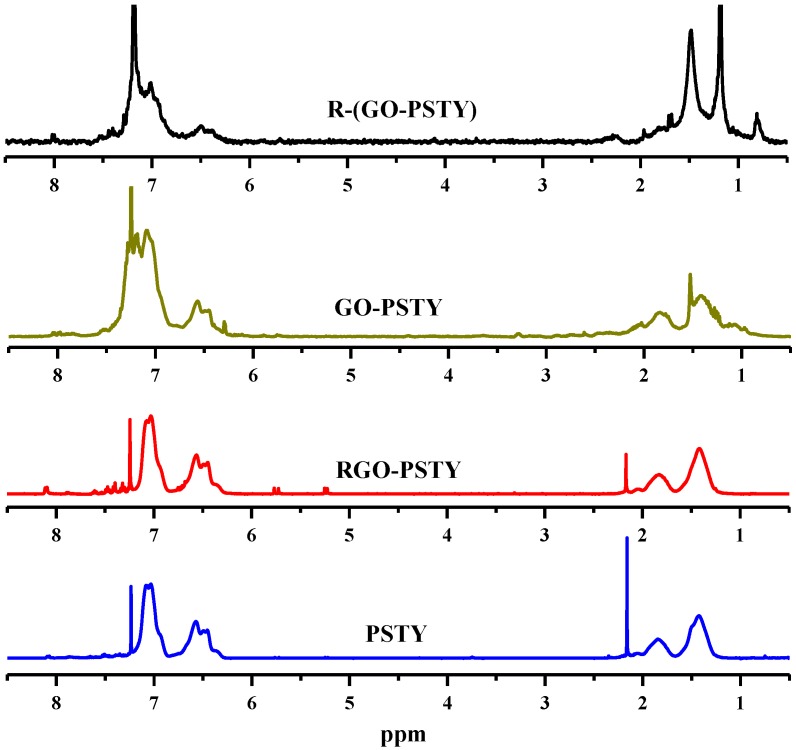
^1^ H-NMR spectra of neat PSTY and all of the composites prepared from *in situ* bulk polymerization and microwave irradiation (MWI).

Additional structural evidence can be obtained from ^1^ H-NMR. The ^1^ H-NMR spectra of the neat PSTY, RGO-PSTY, GO/PSTY (before reduction) and R-(GO-PSTY) composites are shown in Figure 2. For neat PSTY, the multiplets peaks at δ~6.3–7.2 ppm are due to the resonance of the aromatic phenyl group protons. The peaks at δ~1.0–2.0 ppm are due to the methane and methylene groups protons. The RGO/PSTY composite spectra show the same characteristic peaks as PSTY. Based on the ^1^ H-NMR and FTIR results, it is confirmed that we have successfully synthesized RGO/PSTY composites.

**Figure 3:**
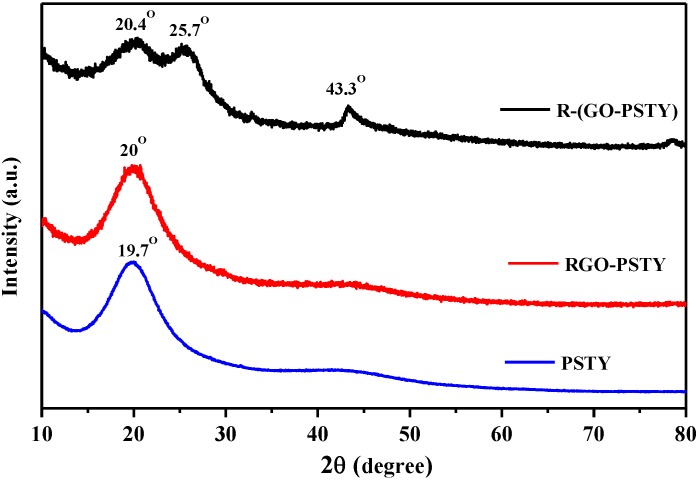
XRD patterns of a neat PSTY, RGO-PSTY and R-(GO-PSTY) composites.

To study the structural changes and exfoliation of RGO sheets within the PSTY matrix, XRD analysis of RGO/PSTY composites was conducted. Figure 3 displays the XRD patterns for neat PSTY, RGO-PSTY and R-(GO-PSTY) composites. The characteristic XRD of graphite, GO and RGO have been reported previously by the authors [20,24]. The XRD pattern of PSTY shows two broad peaks at 2θ of 19.7° and 43.5°, indicating an amorphous structure. In the case of RGO-PSTY composites, the XRD pattern had a broad peak, with a 2θ of 20°, indicating an amorphous structure, which corresponds primarily with the PSTY. However, the XRD pattern of R-(GO-PSTY) composites shows a higher angle by 0.7° and 0.4° compared to PSTY, and RGO-PSTY, respectively. This shift in the diffraction angle in the R-(GO-PSTY) composite to a higher angle indicates that the interlayer spacing decreased due to the extent of exfoliation, which may suggests that the interaction levels between the RGO and the polymer have been enhanced by MWI. In addition, the absence of the diffraction peaks of RGO (12.4°) in the patterns of the composites indicates that the RGO layered structure may change to an exfoliated structure in the composites [27,28,29].

**Figure 4:**
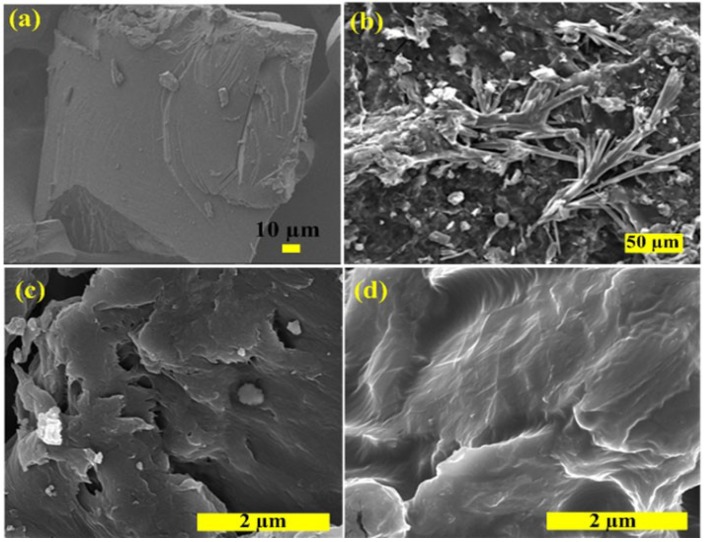
The SEM micrographs of (**a**) neat PSTY; (**b**) GO-PSTY; (**c**) RGO-PSTY and (**d**) R-(GO-PSTY) composites.

Direct evidence for the exfoliation of the RGO in the final polymer composites can be obtained from SEM and HRTEM, which can also provide images of the dispersion in the RGO sheets within PSTY matrix. Figure 4a shows the SEM image of neat PSTY; the image shows a smooth surface. The GO-PSTY composite (Figure 4b) shows the distribution of aggregated GO in the PSTY matrix as branched aggregates reflecting the poor dispersion of GO (hydrophilic) within the PSTY (hydrophobic) matrix. In the case of RGO-PSTY composites prepared by the second approach, Figure 4c shows that RGO sheets are dispersed within the PSTY matrix with a fractured structure. In the case of R-(GO-PSTY) composites (Figure 4d), the wrinkled and smooth profile of RGO sheets is dispersed well within the PSTY matrix. The SEM images of RGO-PSTY and R-(GO-PSTY) composites are quite different, indicating a favorable interfacial interaction of RGO sheets within the PSTY matrix, suggesting a better dispersion and good morphology when MWI is used.

**Figure 5:**
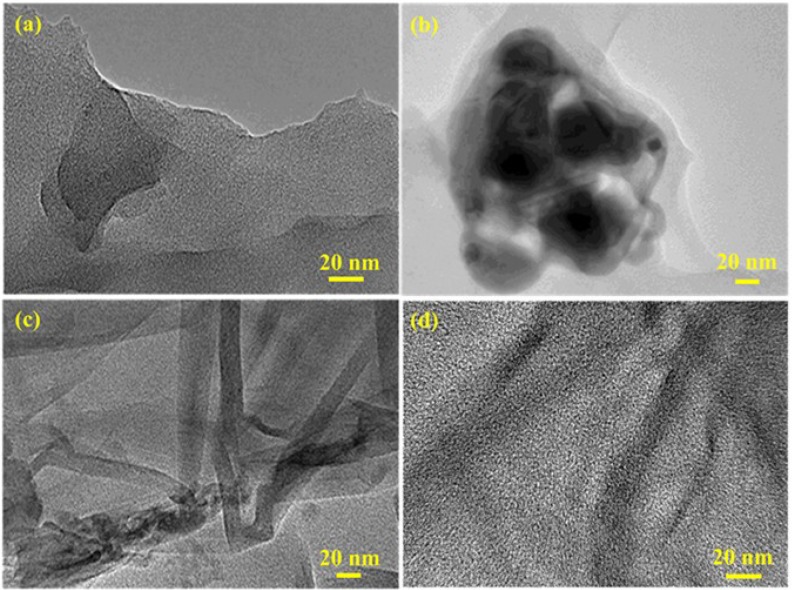
The HR-TEM micrographs of (**a**) neat PSTY; (**b**) GO-PSTY; (**c**) RGO-PSTY and (**d**) R-(GO-PSTY) composites.

High resolution transmission electron microscopy (HRTEM) was employed to determine if the RGO-based sheets were indeed present in the composites as single exfoliated sheets or as multi-layered sheets. Figure 5 displays the HRTEM images of all of the composites and confirms the presence of RGO sheets within the PSTY matrix. The HRTEM image of RGO-PSTY (Figure 5c) shows closely-packed black lines evident in the image, which discloses the presence of multilayered RGO sheets in the composites. These black lines are not ideally aligned in parallel, suggesting that the multilayered RGO sheets are actually composed of restacked single layers [30]. Comparatively, in the case of R-(GO-PSTY), as shown in Figure 5d, it is clearly shown that exfoliated RGO sheets (dark lines) are better uniformly dispersed and ideally aligned in parallel within the PSTY matrix. All in all, the XRD, SEM and HRTEM results suggest that the RGO sheets were exfoliated and dispersed in the PSTY matrix, which indicates good compatibility between the RGO nanosheets and the polymer matrix. This result may be attributed to the covalent interactions between the RGO nanosheets and the PSTY matrix through π–π stacking [31].

**Figure 6:**
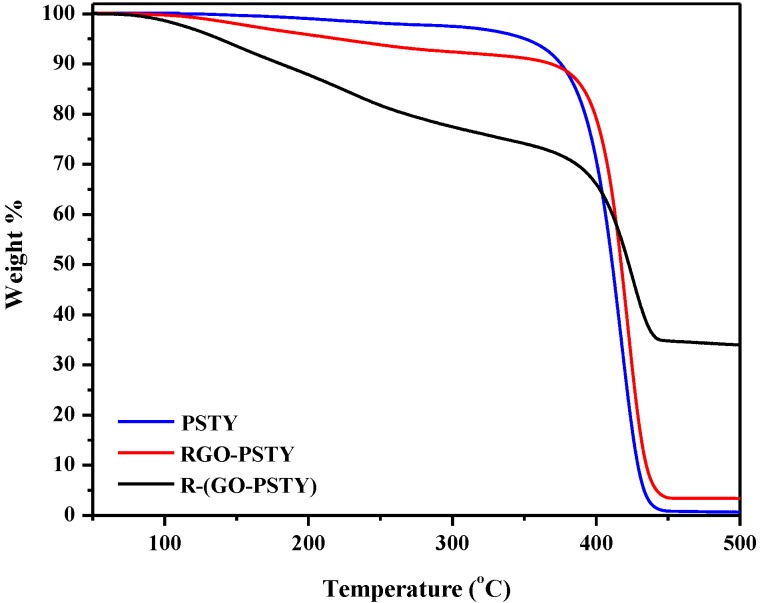
TGA thermograms of neat PSTY, RGO-PSTY and R-(GO-PSTY) composites.

To investigate the effect of the extent of interaction between the PSTY matrix and RGO on the composite properties, we compared the thermal degradation of the neat PSTY with the RGO/PSTY composites. The thermal degradation studies of the neat PSTY, RGO-PSTY and R-(GO-PSTY) composites were performed using thermogravimetric analysis (TGA) under a N_2_ atmosphere. The results are displayed in Figure 6 and summarized in Table 1. The TGA results of the RGO-PSTY composites showed an increase of 5 °C in the maximum degradation temperature (*T*_d_) from 417 to 422 °C compared with the neat PSTY. For the R-(GO-PSTY) composites that were produced using the MWI (Figure 6), the curve shows a maximum degradation temperature (*T*_d_ = 427 °C) that was 10 and 5 °C higher than that of neat PSTY and RGO-PSTY composite, respectively. We attributed this result to the homogenous dispersion and random alignment of the RGO filler within the PSTY matrix. Therefore, incorporation of RGO by MWI can improve the thermal stability of the composites [32].

**Table 1:** Summary of the thermal behavior data obtained from TGA and DSC measurements.

Sample	^a^ *T*_d_ (°C)	^b^ *T*_g_ (°C)	*M*_w_ (g/mol)	*M*_n_ (g/mol)	Polydispersity Index (PDI) ( *M*_w_/*M*_n_)
PSTY	417	90	98,213	33,326	2.95
RGO-PSTY	422	114.9	297,213	135,279	2.20
R-(GO-PSTY)	427	116.3	1.217 × 10^7^	4.095 × 10^6^	2.97

^a^ The maximum degradation temperature in the decomposition stage obtained from the DrTG (derivative thermogram); ^b^ mid-point temperature of *T*_g_.

To further understand the thermal behavior and homogeneity of the composites prepared by the two different methods, differential scanning calorimetry (DSC) of the neat PSTY, RGO-PSTY and R-(GO-PSTY) composites was employed to compare the glass transition temperature (*T*_g_) of the polymer itself to the RGO-PSTY and R-(GO-PSTY) composites.

**Figure 7:**
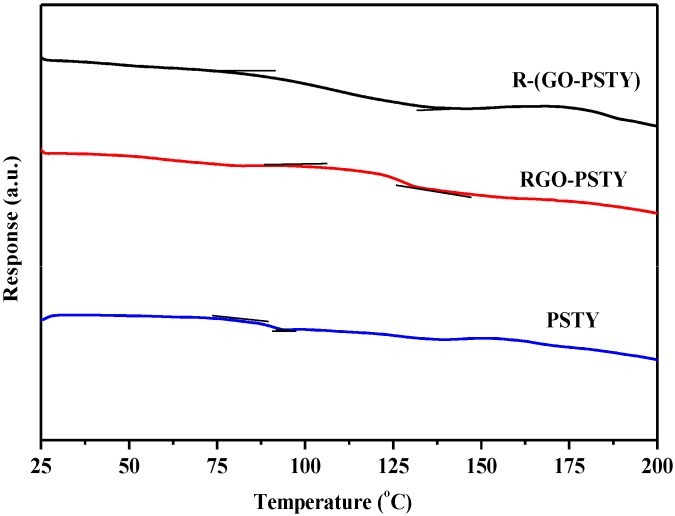
DSC thermograms of neat PSTY, RGO-PSTY and R-(GO-PSTY) composites.

The *T*_g_ values obtained from the DSC thermograms are displayed in Figure 7 and summarized in Table 1. For the RGO-PSTY composite (Figure 7b), the results showed that the *T*_g_ value of the composites after loading the 2% RGO significantly increased by 24.9 °C, which is higher than that of the neat PSTY (*T*_g_ = 90 °C) (Figure 7a). However, when we used the second approach in which the composites were prepared using MWI, the R-(GO-PSTY) composites (Figure 7c) showed a significantly improved thermal stability with a (*T*_g_ = 116.3 °C), which is 1.4 °C higher than that of the RGO-PSTY composites and 26.3 °C higher than that of the neat PSTY, with a broadened transition region. These results suggest a strong interaction between the PSTY matrix and RGO sheets. Previous work has shown that the interfacial strength between nanofillers and polymers, and, consequently, the thermal properties of composites, can be altered by varying the sample preparation method [16,21,33]. In this work, when MWI was used, the *T*_g_ increased upon the addition of the RGO nanofiller, which may be due to either restriction in the chain mobility, resulting from the confinement effect of the 2D-layered RGO incorporated into the matrix, or a strong nanofiller-polymer interaction through π–π interactions. This may be due to the MWI effect, which disrupts the van der Waals interactions and enlarges the interlayer distance of the nanosheets. Therefore, good dispersion without agglomeration of RGO may result from the fast thermal reduction process that is offered by MWI.

When the *in situ* bulk polymerization was used to prepare the RGO-PSTY composites (without MWI), the *T*_g_ also increased by 24.9 °C, which may also be associated with the dispersion of the RGO nanosheets within the PSTY matrix, which may due to the π–π stacking between the π-bond of RGO and the π-bond of styrene, leading to a decrease in the mobility of the polymer chain, resulting from a decrease in the free volume, which has been observed previously [5,23].

The gel permeation chromatography (GPC) results for the RGO/PSTY composites with and without using the MWI are summarized in Table 1. In both cases, the number-average molecular weight (*M*_w_) increased by the addition of RGO. This might suggest that the π-bonds of PSTY and RGO sheets opened by MWI, hence inducing more electron chain transfer sites, thus increasing the average molecular weight (*M*_w_) [32]. Moreover, in the case of R-(GO-PSTY), there is an increase in the polydispersity index (PDI) compared to RGO-PSTY, and this is the same for PSTY, indicating good compatibility and homogeneity between the RGO filler and the PSTY matrix, enhanced by the MWI.

## 3. Experimental Section

### 3.1. Materials

Extra pure graphite powder (>99.5%) was purchased from Merck (Darmstadt, Germany), and hydrazine hydrate (HH, 80%) was obtained from Loba Chemi. Pvt. Ltd. (Mumbai, India). Styrene (STY) (Acros Chemical Co., Loughborough, UK, 99%) was kept in a refrigerator and used as received. Benzoyl peroxide (BP) (BDH Chemicals Ltd., East Yorkshire, UK) was used as an initiator. Other solvents and chemicals were of analytical grade and used without further purification.

### 3.2. Preparation of the Reduced Graphene Oxide (RGO)

The GO powder (400 mg) was stirred and sonicated in deionized water (20 mL) until a homogeneous yellow dispersion was obtained. Then, the solution was placed inside a conventional microwave after the addition of the hydrazine hydrate (HH) reducing agent (40 μL). The microwave oven was operated at full power (900 W) in 30 s cycles (on for 10 s and off and stirring for 20 s), for a total reaction time of 2 min. The yellow dispersion of GO gradually changed to a black color, indicating the completion of the chemical reduction to RGO. The RGO sheets were separated using a centrifuge (Centurion Scientific Ltd., West Sussex, UK) operated at 5000 rpm for 15 min and dried at 80 °C overnight.

### 3.3. Preparation of the RGO-PSTY Composites via the In Situ Method

RGO powder (2.0% wt/wt of STY) prepared via the reduction of GO by MWI, as reported previously [24], was added to the STY monomer, stirred and sonicated for 1 h. The benzoyl peroxide (BP) initiator (5.0 wt% of STY) was added to the suspension and stirred until the initiator dissolved. Then, the mixture was heated to 60 °C to initiate the polymerization using a shaking-water bath (GFL, Burgwedel, Germany). The reaction mixture was maintained at 60 °C for 20 h. After the polymerization finished, the product was poured into an excess of methanol, stirred for 15 min and washed with hot water to remove the unreacted STY monomers. Then, the product was filtered and dried at 80 °C overnight.

### 3.4. Preparation of the R-(GO-PSTY) Composites via the MWI Method

GO powder (2.0% wt/wt of STY) was synthesized from the oxidation of graphite powder via the Hummers and Offeman method [25], then added to the STY monomer, stirred and sonicated for 1 h. The BP initiator (5.0 wt% of STY) was added to the suspension and stirred until the initiator dissolved. Then, the reaction mixture was maintained at 60 °C for 20 h to promote polymerization using a shaking-water bath (GFL). After the polymerization finished, the product was poured into an excess of methanol, stirred for 15 min and washed with hot water to remove the unreacted STY monomers. Then, the product was filtered and dried at 80 °C overnight. Four hundred milligrams of the dried composite of GO-PSTY were dissolved in Toluene solvent, stirred and sonicated for 1 h. Then, the composite was placed inside a conventional microwave oven (Kenwood MW740) following the addition of 40 μL of HH. The microwave oven was operated at full power (900 W) in 30 s cycles (on for 10 s and off and stirring for 20 s) for a total reaction time of 2 min [16]. Then, the composites were separated using a centrifuge (Centurion Scientific Ltd., West Sussex, UK) operated at 5000 rpm for 15 min and dried in an oven at 80 °C overnight.

For comparison, the neat PSTY was prepared via a similar procedure in the absence of the RGO and GO.

### 3.5. Characterization and Instrumentation

The FTIR (Thermo Scientific Nicolet-iS10, Madison, WI, USA) spectra of the composites were recorded in the range of 4000–500 cm^−1^ with KBr pellets. The disks were prepared with a 4% (w/w) sample/KBr powder ratio. The ^1^ H-NMR of the solutions was recorded on a Bruker Avance (III) instrument (Bruker, Milton, ON, Canada) at 200 MHz using deuterated chloroform (CDCl_3_) as a solvent and TMS as an internal standard, and the composites were macerated in the solvent for 1 day. The X-ray diffraction (Philips-Holland, PW 1729, Amsterdam, The Netherlands) of the composites was investigated with Cu radiation (30 kV, 40 mA, Kα radiation (λ = 1.54430 Å)) between 2θ of 5° and 100°. The thermogravimetric analyses (TGA) of the composites were studied using a NETZCH 209 F1 thermogravimetric analyzer (Netzsch, Selb, Germany). The decomposition temperature measurements using TGA were performed under an N_2_ atmosphere at a heating rate of 10 °C per minute from 25 °C to 800 °C. Differential scanning calorimetry (DSC, NETZCH 204 F1) measurements were employed to estimate the glass-transition temperature (*T*_g_) of each composite. The composites were heated from −25 °C to 100 °C at a heating rate of 10 °C per min. Then, a double run was performed after cooling at a heating rate of 2 °C per min from 25 °C to 350 °C. The *T*_g_ was taken as the midpoint of the transition. A scanning electron microscope (SEM, FEI Quanta 200, FEI, Hillsboro, OR, USA) was employed to study the morphology of the composites after they were mounted on the composite slabs and coated with gold via a sputtering system (Polaron E6100, Bio-Rad, Birmingham, UK). Ultrathin sections of the composites were prepared for transmission electron microscopy (TEM) studies; the transmission electron microscope (JEOL JSM-2100F, JEOL, Tokyo, Japan) was operated at 200 kV. Average molecular weight (*M*w) and weight average (*M*n) molecular weight of composites were determined by GPC (HT-GPC Module 350A, Viscotek, Houston, TX 77060, USA. GPC equipped with CLM6210 HT-GPC column) at 35 °C; the flow rate of the carrier solvent, tetrahydrofuran (THF), was 1.0 mL/min.

## 4. Conclusions

Polystyrene containing 2% RGO (PSTY/RGO) composites was successfully prepared by *in situ* bulk polymerization via MWI. Experimental results by X-ray diffraction (XRD), Raman spectroscopy and thermogravimetric analysis (TGA) indicated the successful incorporation of the RGO within the polymer matrix. High resolution transmission electron microscopy (HRTEM) results supported the assumption that the incorporation of the RGO nanosheets in (PSTY/RGO) composites was due to the exfoliation of the filler layers. In addition, the results indicate that the composite obtained using our experimental approach, which involved MWI, had a better morphology and dispersion with enhanced thermal stability, compared with the composites prepared without MWI. In addition, the results showed that the *T*_g_ value of the composites after loading the RGO significantly increased by 24.6 °C.

## References

[1] Geim A.K., Novoselov K.S. (2007). The rise of graphene. Nat. Mater..

[2] Dresselhaus M.S., Dresselhaus G. (1981). Intercalation compounds of graphite. Adv. Phys..

[3] Balandin A.A. (2011). Thermal properties of graphene and nanostructured carbon materials. Nat. Mater..

[4] Park S., An J., Jung I., Piner R.D., An S.J., Li X., Velamakanni A., Ruoff R.S. (2009). Colloidal suspensions of highly reduced graphene oxide in a wide variety of organic solvents. Nano Lett..

[5] Wu N., She X., Yang D., Wu X., Su F., Chen Y. (2012). Synthesis of network reduced graphene oxide in polystyrene matrix by a two-step reduction method for superior conductivity of the composite. J. Mater. Chem..

[6] Singh V., Joung D., Zhai L., Das S., Khondaker S.I., Seal S. (2011). Graphene based materials: Past, present and future. Prog. Mater. Sci..

[7] Huang X., Yin Z.Y., Wu S.X., Qi X.Y., He Q.Y., Zhang Q.C., Yan Q.Y., Boey F., Zhang H. (2011). Graphene-based materials: Synthesis, characterization, properties, and applications. Small.

[8] Paul D.R., Robeson L.M. (2008). Polymer nanotechnology: Nanocomposites. Polymer.

[9] Kuilla T., Bhadra S., Yao D., Kim N.H., Bose S., Lee J.H. (2010). Recent advances in graphene based polymer composites. Prog. Polym. Sci..

[10] Shen B., Zhai W., Chen C., Lu D., Wang J., Zheng W. (2011). Melt blending *in situ* enhances the interaction between polystyrene and graphene through π–π stacking. ACS Appl. Mater. Interfaces.

[11] Potts J.R., Dreyer D.R., Bielawski C.W., Ruoff R.S. (2011). Graphene-based polymer nanocomposites. Polymer.

[12] Wang J., Hu H., Wang X., Xu C., Zhang M., Shang X. (2011). Preparation and mechanical and electrical properties of graphene nanosheets-poly(methyl methacrylate) nanocomposites via *in situ* suspension polymerization. J. Appl. Polym. Sci..

[13] Stankovich S., Dikin D.A., Dommett G.H.B., Kohlhaas K.M., Zimney E.J., Stach E.A., Piner R.D., Nguyen S.T., Ruoff R.S. (2006). Graphene-based composite materials. Nature.

[14] Eda G., Chhowalla M. (2009). Graphene-based composite thin films for electronics. Nano Lett..

[15] El-Shall M.S., Abdelsayed V., Khder A.E.R.S., Hassan H.M.A., El-Kaderi H.M., Reich T.E. (2009). Metallic and bimetallic nanocatalysts incorporated into highly porous coordination polymer MIL-101. J. Mater. Chem..

[16] Patole A.S., Patole S.P., Kang H., Yoo J.-B., Kim T.-H., Ahn J.-H. (2010). A facile approach to the fabrication of graphene/polystyrene nanocomposite by *in situ* microemulsion polymerization. J. Colloid Interface Sci..

[17] Hassan H.M.A., Abdelsayed V., Khder A., AbouZeid K.M., Terner J., El-Shall M.S., Al-Resayes S.I., El-Azhary A.A. (2009). Microwave synthesis of graphene sheets supporting metal nanocrystals in aqueous and organic media. J. Mater. Chem..

[18] Zedan A.F., Sappal S., Moussa S., El-Shall M.S. (2010). Ligand-controlled microwave synthesis of cubic and hexagonal cdse nanocrystals supported on graphene. Photoluminescence quenching by Graphene. J. Phys. Chem. C.

[19] Siamaki A.R., Khder A.E.R.S., Abdelsayed V., el-Shall M.S., Gupton B.F. (2011). Microwave-assisted synthesis of palladium nanoparticles supported on graphene: A highly active and recyclable catalyst for carbon–carbon cross-coupling reactions. J. Catal..

[20] Aldosari M., Othman A., Alsharaeh E. (2013). Synthesis and characterization of the *in situ* bulk polymerization of pmma containing graphene sheets using microwave irradiation. Molecules.

[21] Hu H., Wang X., Wang J., Wan L., Liu F., Zheng H., Chen R., Xu C. (2010). Preparation and properties of graphene nanosheets-polystyrene nanocomposites via *in situ* emulsion polymerization. Chem. Phys. Lett..

[22] Zhang L., Shi T., Wu S., Zhou H. (2014). Graphene/polystyrene nanocomposites synthesized via pickering emulsion polymerization. High Perform. Polym..

[23] Wu X., Liu P. (2010). Facile preparation and characterization of graphene nanosheets/polystyrene composites. Macromol. Res..

[24] Alsharaeh E.H., Faisal N.H., Othman A.A., Ahmed R. (2013). Evaluation of nanomechanical properties of (styrene-methyl methacrylate) copolymer composites containing graphene sheets. Ind. Eng. Chem. Res..

[25] Hummers W.S., Offeman R.E. (1958). Preparation of graphitic oxide. J. Am. Chem. Soc..

[26] Yin G., Zheng Z., Wang H., Du Q., Zhang H. (2013). Preparation of graphene oxide coated polystyrene microspheres by pickering emulsion polymerization. J. Colloid Interface Sci..

[27] Dreyer D.R., Park S., Bielawski C.W., Ruoff R.S. (2010). The chemistry of graphene oxide. Chem. Soc. Rev..

[28] Subrahmanyam K.S., Vivekchand S.R.C., Govindaraj A., Rao C.N.R. (2008). A study of graphenes prepared by different methods: Characterization, properties and solubilization. J. Mater. Chem..

[29] Cao Y., Feng J., Wu P. (2010). Preparation of organically dispersible graphene nanosheet powders through a lyophilization method and their poly(lactic acid) composites. Carbon.

[30] Long J., Chen G. (2013). Fabrication of polymer/microwave-reduced graphite oxide nanocomposites by ball-milling assisted wet compounding. Polym. Compos..

[31] Georgakilas V., Otyepka M., Bourlinos A.B., Chandra V., Kim N., Kemp K.C., Hobza P., Zboril R., Kim K.S. (2012). Functionalization of graphene: Covalent and non-covalent approaches, derivatives and applications. Chem. Rev..

[32] Lee S.H., Dreyer D.R., An J., Velamakanni A., Piner R.D., Park S., Zhu Y., Kim S.O., Bielawski C.W., Ruoff R.S. (2010). polymer brushes via controlled, surface-initiated atom transfer radical polymerization (atrp) from graphene oxide. Macromol. Rapid Commun..

[33] Tang Z., Lei Y., Guo B., Zhang L., Jia D. (2012). The use of rhodamine B-decorated graphene as a reinforcement in polyvinyl alcohol composites. Polymer.

